# Safety of Obtaining an Extra Biobank Kidney Biopsy Core

**DOI:** 10.3390/jcm11051459

**Published:** 2022-03-07

**Authors:** Sheila Bermejo, Clara García-Carro, Richard Mast, Ander Vergara, Irene Agraz, Juan Carlos León, Monica Bolufer, Maria-Alejandra Gabaldon, Daniel Serón, Oriol Bestard, Maria Jose Soler

**Affiliations:** 1 Nephrology Department, Hospital de Vall d’Hebron, 08035 Barcelona, Spain; avergara@vhebron.net (A.V.); iagraz@vhebron.net (I.A.); j.leon@vhebron.net (J.C.L.); mbolufer@vhebron.net (M.B.); dseron@vhebron.net (D.S.); obestard@vhebron.net (O.B.); 2 Nephrology Department, Hospital Clínico San Carlos, 28940 Madrid, Spain; cgcarro@salud.madrid.org; 3 Radiology Department, Hospital de Vall d’Hebron, 08035 Barcelona, Spain; rmast@vhebron.net; 4 Pathology Department, Hospital de Vall d’Hebron, 08035 Barcelona, Spain; magabaldon@vhebron.net

**Keywords:** kidney biopsy, complications, chronic kidney disease, biobank

## Abstract

Background and objectives: Kidney biopsy (KB) is the “gold standard” for the diagnosis of nephropathies and it is a diagnostic tool that presents a low rate of complications. Nowadays, biobank collections of renal tissue of patients with proven renal pathology are essential for research in nephrology. To provide enough tissue for the biobank collection, it is usually needed to obtain an extra kidney core at the time of kidney biopsy. The objective of our study is to evaluate the complications after KB and to analyze whether obtaining an extra core increases the risk of complications. Material and methods: Prospective observational study of KBs performed at Vall d’Hebron Hospital between 2019 and 2020. All patients who accepted to participate to our research biobank of native kidney biopsies were included to the study. Clinical and laboratory data were reviewed and we studied risk factors associated with complications. Results: A total of 221 patients were included, mean age 56.6 (±16.8) years, 130 (58.8%) were men, creatinine was 2.24 (±1.94) mg/dL, proteinuria 1.56 (0.506–3.590) g/24 h, hemoglobin 12.03 (±2.3) g/dL, INR 0.99 (±0.1), and prothrombin time (PT) 11.86 (±1.2) s. A total of 38 patients (17.2%) presented complications associated with the procedure: 13.1% were minor complications, 11.3% (*n* = 25) required blood transfusion, 1.4% (*n* = 3) had severe hematomas, 2.3% (*n* = 5) required embolization, and 0.5% (*n* = 1) presented arterio-venous fistula. An increased risk for complication was independently associated with obtaining a single kidney core (vs. 2 and 3 cores) (*p* = 0.021). Conclusions: KB is an invasive and safe procedure with a low percentage of complications. Obtaining an extra kidney core for research does not increase the risk of complications during the intervention, which remains low in concordance with previously published reports.

## 1. Introduction

Kidney biopsy (KB) is the “gold standard” technique for the diagnosis of renal pathologies [1]. Approximately 20% of the cardiac output reaches the kidneys, which are highly vascularized and, therefore, the main risk of performing a kidney biopsy is bleeding [2,3]. Biopsies were introduced in clinical practice in the 1950s [4,5]. Over the years, the technique has evolved to ultrasound-guided kidney biopsy, which was introduced in the 1980s and was associated with a significant reduction of the complications [6,7]. However, percutaneous puncture remains an invasive procedure and it is important to assess the pitfalls and benefits in patients at high risk of bleeding [8]. In high-risk patients, several centers perform trans-jugular renal biopsy to reduce the rate of complications [9,10]. However, this technique is often related with a smaller sample of renal tissue [11]. The percentage of complications reported in the literature is low, although there is an important variability in reported complications depending on the analyzed series. [2,12,13,14]. The following are considered minor complications (Table 1): pain in the puncture area, hematuria, or hematoma without the need of blood transfusion. Major complications include the need for post-procedural transfusion, embolization, nephrectomy, or death. 

In general, the previously published series demonstrated minor complications in 10–20% of cases and major complications in 1–6% of KB [15]. Most serious complications (such as embolization, nephrectomy, or death) have been rarely reported, with a percentage of less than 1% [6]. The series of Asian KB reported a lower percentage of blood transfusion in comparison to Europe or the United States, and in Europe, the incidence of macroscopic hematuria is much lower than in the United States and Asia [6]. Most of the published studies include a large number of KBs performed in reference centers and it has been described that a smaller size of the hospital center (which implies the performance of less than 30 renal biopsies per year) is a risk factor of complications [7]. This fact may be in part related to the scarce experience on the KB procedure in these centers, suggesting that perhaps bigger reference centers should centralize this technique. Over the years, several bleeding risk factors have been described in kidney biopsy, although the results are not consistent [16]. Regarding age, both older age [12,17,18] and younger age [13,19,20,21,22] have been described as risk factors for bleeding. Female sex [13,19,20], poor blood pressure control [18,23], low hemoglobin level, increased prothrombin time (PT) [12], and decline in renal function [16,17,21,23,24], especially acute kidney injury in hospitalized patients [6], have also been identified as risk factors for complications. Renal tissue biobanks constitute an important tool to implement research and develop new strategies for the diagnosis and treatment of kidney diseases. However, these collections rely on the obtention of more renal material during the KB procedure. Generally, an extra renal biopsy core is needed for storage, since two cores are normally used for diagnosis. There is some literature about the complications related to the obtention of an extra third renal core for research purposes. Our hypothesis is that obtaining an extra renal tissue core does not increase the rate of complications of KB in patients who agreed to donate kidney material to the nephrology biobank. The aim of this study is to evaluate the percentage and severity of complications in patients with renal disease who accepted to participate at our native kidney research biobank. 

## 2. Material and Methods

### 2.1. Sample Selection

This is a prospective and observational study in which native KB performed at the Vall d’Hebron Hospital from January 2019 to December 2020 have been included. Since January 2019, our hospital has a biobank of native kidney biopsies approved by the Ethical committee for clinical research—Vall d’Hebron institute of Research (CEIC-VHIR) (PR (AG) 252/2018), as well as by the Department of Health of the Generalitat de Catalunya. In our hospital, ultrasound-guided KB is performed by an interventional radiologist in the presence of a clinical nephrologist. The thickness of the needle usually used is 16G and the choice of the side of the kidney biopsy is an individualized decision of the radiologist in each case. Furthermore, the indications of trans-jugular renal biopsy in our center are: patients with combined liver biopsy, a single kidney, or hemostasis alterations that confer a high risk of bleeding. This technique is performed by an angio-radiologist approaching the posterior right inferior renal vein through the right jugular vein. The contraindications of this technique are the presence of an internal jugular vein thrombosis or a serious coagulation disorder that contraindicates the puncture of a central venous access. In general, two kidney cores were obtained and subsequently processed in the pathology department by the usual techniques (optical microscopy and electron microscopy) for histological diagnosis. All study subjects signed the informed consent. An extra third kidney core biopsy was obtained and stored in RNA later solution when possible. RNAlater Solution is an aqueous stabilizing solution that permeates most tissues rapidly to stabilize and protect RNA in fresh samples, eliminating the need for immediate freezing. Renal tissues were stored in RNAlater solution at −20 °C. The inclusion criteria were patients who signed informed consent, older than 18 years old, and the realization of native kidney biopsy. The exclusion criteria were patients who did not signed informed consent, younger than 18 years old, and renal transplant patients.

### 2.2. Clinical Variables

A total of 33 variables were analyzed: 23 (69.7%) clinical and 10 (30.3%) analytical. Demographic variables (sex and age), anthropomorphic characteristics (weight, height, and body mass index (BMI)), medical pathological history (hypertension (defined as >140/90 mmHg) and diabetes mellitus), chronic medication (antiplatelet and/or anticoagulant), analytical variables (serum creatinine level in mg/dL, proteinuria in g/24 h, albumin/creatinine ratio in mg/g and protein/creatinine ratio in mg/g, presence of microhematuria, hemoglobin level in g/dL before and after biopsy, blood count platelets, INR level, and PT in seconds), as well as the histological diagnosis. In addition, the type of technique used for the kidney biopsy (ultrasound-guided vs. trans-jugular), the kidney from which the biopsy was obtained (right vs. left), the number of kidney cores obtained, and kidney and cortical size in centimeters were collected (cm) and systolic and diastolic blood pressure in mmHg were also collected. Finally, the complications derived from the KB were recorded. Complications were classified as minor, corresponding to patients who presented mild hematoma without transfusion requirement or cause prolonged admission. Major complications included patients with severe hematoma that required a longer hospital stay, anemia requiring transfusion, hematuria or hemodynamic instability, need of arterial embolization, arteriovenous fistula, and death. With the purpose of classifying patients into major or minor complications, the most severe complication was the one registered.

### 2.3. Statistical Analysis

For the statistical analysis, the computer program IBM SPSS Statistics version 20.0 was used. The quantitative variables with normal distribution were presented as mean and standard deviation. Variables that do not follow a normal distribution were presented as median and interquartile range. Qualitative variables were expressed as a percentage. For the comparison of qualitative variables, we used the chi square test and for the comparison of the means between two samples, we used the Student’s *t*-test for independent data and U Mann–Whitney test for variables that do not follow a normal distribution. The comparison between means with more than two categories was performed with an ANOVA test and the Tukey test for multiple comparisons between different groups. A binary logistic regression multivariate analysis was performed with the presence of post-kidney biopsy complications as dependent variable. A multinomial logistic multivariate analysis was performed with the presence of major complications, minor complications, and absence of complications as dependent variables. Statistical significance was considered when *p* was less or equal than 0.05.

## 3. Results

### 3.1. Baseline Characteristics of Population

A total of 221 patients were included (Table 2) with a mean age of 56.6 (±16.8) years. 

A total of 130 (58.8%) patients were men, 128 (57.9%) patients had hypertension, and 52 (23.5%) were diabetic. The mean weight was 75.8 (±24.6) kg and a BMI of 27.6 (±5.01) (kg/m^2^), proteinuria 1.56 (0.506–3.59) g/24 h, mean hemoglobin 12.03 (±2.3) g/dL, 250,995 (±89,742) platelets, INR 0.99 (±0.1), PT 11.86 (±1.2) seconds. A total of 100 patients (45.2%) presented microhematuria in the urinary sediment before the KB. A total of 15 (6.8%) patients were under anticoagulation treatment and 36 patients (16.3%) were on antiplatelet treatment. Both anticoagulation and antiplatelet treatments were stopped in all patients at least 5 days before the KB. Regarding the technique used for renal biopsy, in 213 (96.4%) patients it was ultrasound-guided and the remaining *n* = 8 (3.8%) were performed by a transjugular procedure. In 152 (68.8%) patients, three renal tissue cores were obtained, in 62 (28.1%) patients, two cores, and in 7 (3.2%) patients, a single core was obtained. A sample for the biobank was obtained in a total of 180 (81.4%) patients, in 138 cases (76.7%), 3 renal cores were obtained, in 40 patients (22.2%), 2 renal cylinders, and in 2 cases (1.1%) obtaining a unique renal core was sufficient to obtain material for the biobank. Reasons for a research biopsy core not being obtained were operator choice in patients presenting a bleeding complication during the procedure before collection of research tissue (23.2%, *n* = 16). We analyzed and compared the patients’ characteristics according to the number of kidney cores that were obtained in the procedure (Table 3). 

We found differences in the terms of renal function and the side of punction. Thus, patients where one core was obtained presented worse baseline renal function as compared with the patients where two or three cores were obtained (4.5 mg/dL median of serum creatinine vs. 2.5 mg/dL and 2 mg/dL, respectively, *p* = 0.002). Furthermore, in patients with three cores, the left kidney was more frequently biopsied as compared with the right side (77.8% on left kidney biopsy vs. 61.7% on the right kidney, *p* = 0.03).

**Figure 1:**
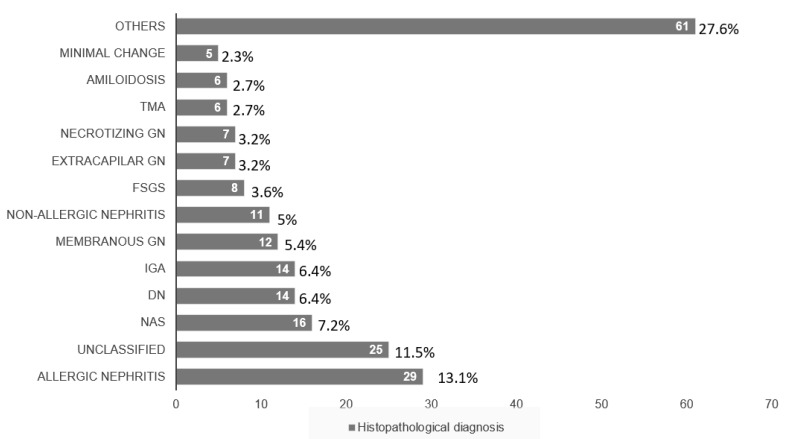
Histopathological diagnostics at kidney biopsy. Distribution in number and percentages of the patients according to their histopathological diagnosis. TMA: Thrombotic Microangiopathy, GN: Glomerulonefritis, FSGS: Focal and segmental glomeruloesclerosis, DN: Diabetic Nephropathy, NAS: Nephroangioesclerosis.

The most frequent histological diagnosis was acute interstitial nephritis (13.1%, *n* = 29), followed by nonspecific diagnosis (11.3%, *n* = 25), nephroangiosclerosis (7.2%, *n* = 16), diabetic nephropathy (6.4%, *n* = 14), IgA nephropathy (6.3%, *n* = 14), membranous nephropathy (5.4%, *n* = 12), and non-allergic interstitial nephritis (5%, *n* = 11) (Figure 1).

### 3.2. Post-Kidney Biopsy Complications

**Figure 2:**
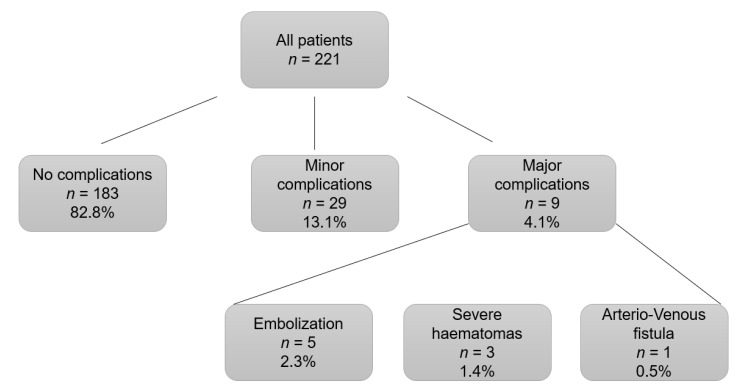
Complications post-kidney biopsy. Distribution by percentages according to presenting complications after kidney biopsy.

A total of 38 (17.2%) complications associated with the procedure were recorded. A total of 11.3% (*n* = 25) of patients required blood transfusion, 12 patients post-KB, and 13 patients pre-KB. The indications of blood transfusion pre-KB were level of hemoglobin < 8 g/dL to ensure a higher level of safety prior to the invasive procedure. A total of 5.3% (*n* = 38) of patients with complications, presented macrohematuria. Most of the complications were minor complications (76.3%, *n* = 29), mild hematomas that represented 13.1% of the total number of kidney biopsies performed (see Figure 2). 

Severe hematomas were diagnosed in 1.4% (*n* = 3), which corresponds to 7.9% of the total complications. A total of five patients (2.3% of all renal biopsies, 13.2% of complications) required embolization and in one case (0.5% of all procedures, 2.6% of complications) the complication was an arterio-venous fistula. We did not observe any cases of nephrectomy or death. The patients who presented complications (major and minor) were classified and compared with those who did not present any type of complication (Table 4). 

We observed that the patients who presented complications were younger (*p* = 0.034), had lower body weight (*p* = 0.022), higher platelet count (*p* = 0.038), lower PT (*p* = 0.05), and required a higher percentage of blood transfusions (*p* = 0.003). A tendency of lower pre-kidney biopsy hemoglobin level was observed in patients who presented complications (*p* = 0.057). There was no evidence of a higher number of complications in patients from whom three renal tissue cores were obtained in comparison to patients with one or two cores (*p* = 0.012). In addition, we also compared the baseline characteristics of the population according to the degree of complications (major, minor, and without complication) (Table 5). 

We found that patients with major complications presented a longer INR and PT (*p* = 0.028 and *p* = 0.02, respectively), they were more likely to have undergone transjugular kidney biopsy (*p* = 0.006), and required a higher number of blood transfusions peri-procedure (*p* < 0.001).

### 3.3. Risk Factors for Complications Associated with Kidney Biopsy

To identify the risk factors for kidney biopsy complications, a multivariate binary logistic regression analysis was performed with the presence of any complication (major or minor) as a dependent variable (Table 6). 

The model was adjusted by variables that were statistically significant in the previous univariate analysis: age, hemoglobin level, number of renal tissue cores, number of platelets, PT, and weight. Obtaining a single kidney core (vs. 2 and 3 cores) was identified as independent risk factors of bleeding (*p* = 0.021). A second multivariate logistic regression model was performed in which the dependent variable was the presence of major complications, adjusting for the same variables as the previous model. However, in this case, no statistically significant risk factors for major complications were identified. Furthermore, we performed another multivariate multinomial analysis for independent risk factors for presenting minor complications vs. major complications vs. absence of complications (Supplementary Table S1). This model was adjusted by the same variables as the previous analysis. We evidenced that higher level of PT (*p* = 0.035) and obtaining single kidney core (*p* = 0.026) were independent risk factors of presenting minor complications. Regarding major complications, we did not find any independent risk factors.

## 4. Discussion

As far as we know, this study analyzes for the first time whether obtaining a third renal tissue core in KB increases the risk of complications. A total of 221 patients accepted to participate in our native kidney research biobank from January 2019 to December 2020. During this time, a kidney core for research purposes was obtained in 68.8% of patients. As patients that underwent a third extra core did not show more complications, we demonstrated that obtaining an extra core KB is safe. In the present study, we evidenced a total of 17.3% complications (major and minor) associated with the procedure. Only 4.1% of the total showed major complications, and it should be noted that no case of nephrectomy or death was detected. In our cohort, obtaining a single kidney core (vs. two and three cores) was identified as an independent risk factor of bleeding.

For the purpose of analyzing the complications after KB, it is important to take into account the protocol of the procedure of each center to be able to interpret correctly the results of the studies. Thus, it is important to consider the thickness of the needle used for the puncture and the number of cores that are usually obtained. Several studies that have been focused on post-kidney biopsy complications detailed these relevant aspects [12,25,26,27,28,29,30,31,32,33,34,35] (Table 7). 

However, in a few studies, the KB protocol details are not explained [13,14,36]. The thickness of the needles used most frequently was 16G or 18G; usually, the choice of the needle size depends on the professional who performs the kidney biopsy [25,27,28,29,30,33]. In other cases, the size of needle that they used is defined by protocol of the center [12,26,31,32,33,34,35]. These last-mentioned studies are in concordance with our center, where 16G needles are used by protocol. The number of kidney cores obtained is also variable. In some studies, the mean of the kidney cores obtained is reported [25,30]; however, in the majority of studies, the percentages of patients from whom a certain number of cores are obtained are not specified [27,28,31,32,33,34,35]. Only a few studies reported the exact percentage of kidney cores obtained [12,26,29]. In these cases, the most frequent number of kidney cores obtained was two. As expected by the nature of our study, three cores were the most frequent number of kidney cores obtained followed by two cores and one core, respectively. In most of the studies, the differences between the number of cores obtained in relation with the risk complications has not been assessed; in some of the studies where it has been calculated, no differences between different core number groups have been found [27,28,32,33].

Previous studies focused on post-kidney biopsy complications obtained similar percentages to those observed in the present study. Pombas et al., in a Spanish cohort of 661 kidney biopsies, diagnosed 16.6% of complications [12]. The percentage of major complications was lower as compared to our study (1.5% vs. 4.1% of KB patients). It is important to highlight that several studies with larger cohorts have also reported a similar percentage of major complications as our study [2,13,14]. Peters et al. analyzed a cohort in Sweden of 2835 kidney biopsies and obtained a percentage of major complications of 5.65%, while Palsson et al. evidenced 4.3% of their patients presented major complications [2,13]. Halimi et al. studied a large French cohort of 52,135 kidney biopsies and showed that the percentage of major complications was 5% [14]. In a recently published meta-analysis, a total of 87 studies were included in which 118,064 kidney biopsies were included; they demonstrated that post-kidney biopsy pain was detected in 4.3%, hematomas in 11%, macroscopic hematuria in 3.5%, bleeding requiring blood transfusion in 1.6%, and intervention requirements for bleeding in 0.3% of KB [6]. In our cohort, there was a higher need of blood transfusion (11.3%), and in 2.4% of the cases, embolization was required. However, the hemoglobin level where the physician indicates a blood transfusion has been not described in the published studies [2,6,12,13,14]. This may in part increase the variability of the different published studies. 

In previous studies, the transfusion requirement was lower, with percentages ranging from 1.2% to 5% [2,6,12,14,24,28]. However, Peters B et al. reported a need of blood transfusion in 23.42% of their cohort, which is higher than that evidenced in the present study [13]; the need of blood transfusion was detected in 11.3% of all patients in our cohort. In previous studies, the transfusion requirement was lower, with percentages ranging from 1.2% to 5% [2,6,12,14,24,28]. However, Peters B et al. reported a need of blood transfusion of 23.42% in their cohort, which is higher than that evidenced in the present study [13]. In the case of blood transfusion requirement or anemia as the risk factor for complications in patients that underwent KB, it is difficult to decipher if it is a risk factor or a worse clinical status of the patient “per se” [37].

Finally, obtaining of a single renal tissue core was also identified as a risk factor for complications as compared to those with two or three kidney cores. A plausible explanation for this finding is that in the patients who presented a bleeding complication, it was mainly detected at the first puncture, and for that reason, only a single kidney core was obtained. Another possible explanation suggested that increased bleeding risk (e.g., low platelets and anticoagulants) per se could have contributed to perform only one biopsy, with more bleeding complications thereafter.

As mentioned above, in our study, obtaining more kidney cores was not identified as a risk factor for complications secondary to KB. A study called TRIDENT (Transforming Research in Diabetic Nephropathy) is currently ongoing, in which a cohort of biopsied diabetic patients with diabetic nephropathy is analyzed; an extra kidney core has been obtained for research studies purposes [38]. To date, 160 patients have been included in the TRIDENT, and in 144 patients, an extra kidney core was obtained. The percentage of complications was similar in patients with an extra core 7% vs. 6%, respectively [28]. Taking all of these results together, an extra core renal biopsy for research purposes seems a safe procedure in patients with kidney disease.

Our study had some limitations. One of the main limitations of our study is that it is a single center study that accounts for a small sample size. For this reason, one can surmise that several variables were not statistically significant in the analysis. Another limitation is that it is an observational prospective study, and it is not a randomized clinical trial (RCT). Thus, enlarging the sample size, performing a multicenter extra renal core biopsy study, or designing an RCT could help to ascertain the security of obtaining extra cores in KB. In addition, other complications risk factors, such as increased bleeding time and specific coagulation factors, have not been assessed.

Kidney biopsy is an essential tool for the diagnosis and management of patients with renal involvement. Performing this procedure is safe thanks to the improvement of the technique over the years, which has achieved a low rate of complications. Furthermore, obtaining an extra renal core for research, which implies one more puncture during the biopsy procedure, did not increase the risk of presenting complications in our study. Future multicenter studies that include a larger cohort are necessary to evaluate the existence of other risk factors for presenting associated complications in this type of population.

## Figures and Tables

**Table 1:** Definitions of major and minor complications.

Major Complications	Minor Complications
Need of transfusion	Pain
Embolization	Hematuria
Nephrectomy	Hematoma without blood transfusion
Death	

**Table 2:** Baseline characteristics of population.

Characteristics	All Patients
Patients (*n*)	221
Age (years)	56.6 (±16.8)
Sex (Women/Men) (*n*, %)	91 (41.2%)/130 (58.8%)
Hypertension (>140/90 mmHg) (*n*, %)	128 (57.9%)
Diabetes Mellitus (*n*, %)	52 (23.5%)
Weight (kg)	75.8 (±24.6)
BMI (kg/m^2^)	27.6 (±5.01)
Systolic blood pressure (mmHg)	136 (±24.6)
Diastolic blood pressure (mmHg)	76 (±15)
Creatinine (mg/dL)	2.24 (±1.94)
Proteinuria (g/24 h)	1.56 (0.506–3.59)
Urinary albumin/creatinine ratio (mg/g)	613 (100.3–2300)
Urinary protein/creatinine ratio (mg/g)	1153.8 (482.8–3156)
Platelets (*n*)	250.995 (±89.742)
INR	0.99 (±0.1)
PT (seconds)	11.86 (±1.2)
Hb pre (g/dL)	12.03 (±2.3)
Hb post (g/dL)	11.3 (±2.3)
Microhematuria (*n*, %)	100 (45.2%)
Kidney size (cm)	11.04 (±1.2)
Cortical size (cm)	1.67 (±0.64)
Technique (US-guided/transjugular) (*n*, %)	213 (96.4%)/8 (3.6%)
Number of renal cores (n, %):	
3	152 (68.8%)
2	62 (28%)
1	7 (3.2%)
Anticoagulants (n, %):	
Acenocoumarol	7 (3.2%)
Heparin	3 (1.4%)
Others	5 (2.3%)
Antiplatelets (n, %):	
ASA 100 mg	30 (13.6%)
ASA 300 mg	1 (0.5%)
Clopidogrel	5 (2.3%)

BMI: Body Mass Index. PT: Prothrombin time. ASA: Acetyl Salicylic acid. Hb: Hemoglobin.

**Table 3:** Baseline characteristics population according with the number of kidney cores obtained at kidney biopsy.

Characteristics	Number of Kidney Cores			*p*
	1	2	3	
Patients (*n*, %)	7 (3.2%)	62 (28.1%)	151 (68.8%)	-
Age (years)	45.4 (±17.9)	56 (±17.2)	57.4 (±16.5)	0.171
Sex (Women/Men) (*n*, %)	2 (28.6%)/ 5 (71.4%)	29 (46.8%)/ 33 (53.2%)	60 (39.5%)/ 92 (60.5%)	0.486
Hypertension (*n*, %)	5 (71.4%)	37 (59.7%)	86 (56.6%)	0.699
Diabetes Mellitus (*n*, %)	0 (0%)	15 (24.2%)	37 (24.3%)	0.329
Weight (kg)	68.3 (±19.2)	74.8 (±15.3)	76.6 (±15.3)	0.321
BMI (kg/m^2^)	25.4 (±2.8)	27.1 (±5)	27.9 (±5.1)	0.298
Systolic blood pressure (mmHg)	151.3 (±25.9)	134.3 (±28.2)	135.4 (±22.8)	0.22
Diastolic blood pressure (mmHg)	87.7 (±19.2)	76.6 (±15.5)	75.2 (±15)	0.101
Creatinine (mg/dL)	4.5 (±3.3)	2.5 (±2.3)	2 (±1.6)	**0.002**
Proteinuria (g/24 h)	2.8 [2.3–7.8]	3 [2–4]	2.8 [2.2–3.4]	0.948
Urinary albumin/creatinine ratio (mg/g)	387.4 [51.7–826.5]	1577.2 [950–2204.4]	1616 [1243–1989.1]	0.403
Urinary protein/creatinine ratio (mg/g)	131.1 [57.2–2005.1]	2730.8 [1757.9–3703.6]	2740.6 [2080.6–3400.7]	0.619
Platelets (*n*)	260,428 (±58,266)	238,725 (±103,365)	255,565 (±84,828)	0.444
INR	0.99 (±0.1)	0.99 (±0.1)	0.98 (±0.1)	0.807
PT (seconds)	11.9 (±1.2)	11.9 (±1.1)	11.8 (±1.2)	0.843
Hb pre (g/dL)	12.5 (±2.6)	11.7 (±2.4)	12.1 (±2.3)	0.466
Hb post (g/dL)	11.4 (±2.7)	11.1 (±2.6)	11.4 (±2.1)	0.784
Microhematuria (*n*. %)	4 (57.1%)	26 (41.9%)	70 (46.4%)	0.689
Kidney size (cm)	10.7 (±1.2)	11.2 (±1.3)	11 (±1.2)	0.449
Cortical size (cm)	1.7 (±0.2)	1.7 (±0.3)	1.7 (±0.3)	0.985
Left kidney/Right kidney (*n*, %)	3 (42.9%)/ 4 (57.1%)	19 (31.1%)/ 42 (68.9%)	77 (51%)/ 74 (49%)	**0.031**
Technique (US-guided/ Transjugular) (*n*, %)	7 (100%)/ 0	58 (93.5%)/ 4 (6.5%)	148 (97.4%)/ 4 (2.6%)	0.348
Transfusion (*n*, %)	2 (28.6%)	9 (14.5%)	14 (9.2%)	0.184
Anticoagulants (*n*. %):				
Acenocoumarol	0	3 (4.8%)	4 (2.6%)	0.701
Heparin	0	1 (1.6%)	2 (1.3%)	
Others	0	3 (4.8%)	2 (1.3%)	
Antiplatelets (*n*. %):				
ASA 100 mg	0	6 (9.7%)	24 (15.8%)	
ASA 300 mg	0	0	1 (0.7%)	0.743
Clopidogrel	0	2 (3.2%)	3 (2%)	

BMI: Body Mass Index. PT: Prothrombin time. ASA: Acetyl Salicylic acid. Hb: Hemoglobin. *p*-value < 0.05 are in bold.

**Table 4:** Baseline characteristics of patients according to the complications.

Characteristics	Without Complications	With Complications	*p*
Patients (*n*, %)	183 (82.8%)	38 (17.2%)	-
Age (years)	57.7 (±16.3)	51.4 (±18.2)	**0.034**
Sex (Women/Men) (*n*, %)	73 (39.9%)/ 110 (40.1%)	18 (47.4%)/ 20 (52.6%)	0.469
Hypertension (*n*, %)	108 (59%)	20 (52.6%)	0.476
Diabetes Mellitus (*n*, %)	10 (26.3%)	42 (23%)	0.676
Weight (kg)	76.9 (±15.4)	70.5 (±14.5)	**0.022**
BMI (kg/m^2^)	27.8 (±4.97)	26.2 (±5.1)	0.096
Systolic blood pressure (mmHg)	134.5 (±23.8)	141 (±27.5)	0.134
Diastolic blood pressure (mmHg)	75.5 (±15.6)	78.5 (±14.2)	0.262
Creatinine (mg/dL)	2.26 (±1.9)	2.15 (±1.97)	0.751
Proteinuria (g/24 h)	2.85 (2.27–3.43]	3.01 (1.88–4.15]	0.809
Urinary albumin/creatinine ratio (mg/g)	1556.57 (1214.84–1898.31]	1622.96 (856.65–2389.27]	0.875
Urinary protein/creatinine ratio (mg/g)	2653.11 (2065.71–3240.52]	2907.7 (1613.22–4202.19]	0.725
Platelets (*n*)	245,295 (±84,011)	278,447 (±110,613)	**0.038**
INR	0.99 (±0.1)	0.96 (0.09)	0.07
PT (seconds)	11.92 (±1.22)	11.54 (±1.08)	**0.05**
Hb pre (g/dL)	12.07 (±2.27)	11.84 (±2.56)	0.057
Hb post (g/dL)	11.4 (±2.2)	10.8 (±2.5)	0.14
Microhematuria (*n*, %)	84 (46.2%)	16 (42.1%)	0.722
Kidney size (cm)	11.08 (±1.25)	10.84 (±1.09)	0.277
Cortical size (cm)	1.67 (±0.69)	1.64 (±0.32)	0.807
Left kidney/Right kidney (*n*, %)	85 (47%)/ 96 (53%)	24 (63.2%)/ 14 (36.8%)	0.286
Technique (US-guided/transjugular) (*n*, %)	177 (96.7%)/ 6 (3.3%)	36 (94.7%)/ 2 (5.3%)	0.628
Transfusion (*n*, %)	15 (8.2%)	10 (26.3%)	**0.003**
Number of renal cores			
(*n*, %):			
3	130 (71%)	22 (57.9%)	
2	50 (27.3%)	12 (31.6%)	**0.012**
1	3 (1.6%)	4 (10.5%)	
Anticoagulants (*n*, %):			
Acenocoumarol	7 (3.8%)	0	
Heparin	3 (1.6%)	0	0.342
Others	5 (2.7%)	0	
Antiplatelets (*n*, %):			
AAS 100 mg	24 (13.1%)	6 (15.8%)	
AAS 300 mg	1 (0.5%)	0	0.935
Clopidogrel	4 (2.2%)	1 (2.6%)	

BMI: Body Mass Index. PT: Prothrombin time. ASA: Acetyl Salicylic acid. Hb: Hemoglobin, US: Ultrasound. *p*-value < 0.05 are in bold.

**Table 5:** Baseline characteristics of patients according to presenting complications: major, minor, or without complications.

Characteristics	Without Complications	Minor Complications	Major Complications	*p*
Patients (*n*, %)	183 (82.8%)	29 (13.1%)	9 (4.1%)	-
Age (years)	57.7 (±16.3)	52.9 (±17.8)	46.6 (±20)	0.065
Sex (Women/Men) (*n*, %)	73 (39.9%)/ 110 (40.1%)	13 (44.8%)/ 16 (55.2%)	5 (55.6%)/ 4 (44.4%)	0.591
Hypertension (*n*, %)	108 (59%)	17 (58.6%)	3 (33.3%)	0.312
Diabetes Mellitus (*n*, %)	10 (26.3%)	9 (31%)	1 (11.1%)	0.425
Weight (kg)	76.9 (±15.4)	71.04 (±14.4)	68.97 (±15.3)	0.069
BMI (kg/m^2^)	27.8 (±4.97)	26.93 (±5.36)	24.13 (±3.73)	0.097
Systolic blood pressure (mmHg)	134.5 (±23.8)	144.24 (±24.5)	130.67 (±35.4)	0.114
Diastolic blood pressure (mmHg)	75.5 (±15.6)	79.24 (±13.9)	76.22 (±16)	0.468
Creatinine (mg/dL)	2.26 (±1.9)	1.93 (±1.65)	2.86 (±2.76)	0.432
Proteinuria (g/24 h)	2.85 (2.27–3.43)	2.85 (1.57–4.2)	3.47 (0.55–6.39)	0.896
Urinary albumin/creatinine ratio (mg/g)	1556.57 (1214.84–1898.31)	1412.1 (789.6–2034.6)	2255.6 (671.1–5182.3)	0.632
Urinary protein/creatinine ratio (mg/g)	2653.11 (2065.71–3240.52)	2536.5 (1332–3741.02)	4114.2 (536.9–8765.2)	0.562
Platelets (*n*)	245,295 (±84,011)	270,517 (±80,529)	304,000 (±181,464)	0.072
INR	0.99 (±0.1)	0.94 (±0.07)	1.02 (±0.11)	**0.028**
PT (seconds)	11.92 (±1.22)	11.33 (±0.94)	12.2 (±1.29)	**0.032**
Hb pre (g/dL)	12.07 (±2.27)	12.26 (±2.67)	10.49 (±1.64)	0.115
Hb post (g/dL)	11.4 (±2.2)	11.28 (±2.45)	9.24 (±2.13)	0.02
Microhematuria (*n*, %)	84 (46.2%)	12 (41.4%)	4 (44.4%)	0.89
Kidney size (cm)	11.08 (±1.25)	10.93 (±1.1)	10.57 (±1.1)	0.413
Cortical size (cm)	1.67 (±0.69)	1.6 (±0.32)	1.75 (±0.33)	0.829
Left kidney/Right kidney (*n*, %)	85 (47%)/ 96 (53%)	10 (34.5%)/ 19 (65.5%)	4 (44.4%)/ 5 (55.6%)	0.455
Technique (US-guided/ Transjugular) (*n*, %)	177 (96.7%)/ 6 (3.3%)	29 (100%)/ 0	7 (77.8%)/ 2 (22.2%)	**0.006**
Transfusion (*n*, %)	15 (8.2%)	5 (17.2%)	5 (55.6%)	**<0.001**
Number of renal cores (*n*, %):				
3	130 (71%)	17 (58.6%)	5 (55.6%)	
2	50 (27.3%)	9 (31%)	3 (33.3%)	0.064
1	3 (1.6%)	3 (10.3%)	1 (11.1%)	
Anticoagulants (*n*. %):				
Acenocoumarol	7 (3.8%)	0	0	
Heparin	3 (1.6%)	0	0	0.765
Others	5 (2.7%)	0	0	
Antiplatelets (*n*. %):				
ASA 100 mg	24 (13.1%)	6 (20.7%)	0	
ASA 300 mg	1 (0.5%)	0	0	0.378
Clopidogrel	4 (2.2%)	0	1 (11.1%)	

BMI: Body Mass Index. PT: Prothrombin time. ASA: Acetyl Salicylic acid. Hb: Hemoglobin, US: Ultrasound. *p*-value < 0.05 are in bold.

**Table 6:** Multivariate logistic binary regression analysis for independent risk factors of presenting complications after kidney biopsy.

Variable	OR	CI (95%)	Lateral Significance (*p*)
PT (seconds) *	1.36	0.94–1.97	0.1
Renal cores (1 core vs. 2/3 cores)	7.09	1.34–37.04	**0.021**
Age (years) *	1.01	0.99–1.04	0.299
Platelets (*n*) *	1	1–1	0.130
Weight (kg) *	1.02	0.99–1.04	0.245
Hb pre (g/dL) *	1.13	0.94–1.36	0.2

Dependent variable: Complications after kidney biopsy. *PT*: Prothrombin time, Hb pre: Hemoglobin level pre-KB, CI: Confidence Interval, * Quantitative variables included in the multivariate logistic binary regression model. *p*-value < 0.05 are in bold.

**Table 7:** Principal studies for kidney biopsy complications, including technical details.

Study	Country	Year	Type of Study	Number of Patients	Type of Needles	Number of Kidney Cores	Complications Regarding Number of Kidney Cores
Singh S, et al. [25]	United States	2021	Prospective	20	18G (95%) 16G (5%)	From 2 to 6 cores (2.9)	No data
Manno et al. [26]	Italy	2011	Randomized Clinical Trial	162	16G	2 cores	No data
Peters et al. [27]	Sweden	2018	Prospective	576	16G or 18G	From 2 to 3 cores (Unknown)	No differences
Pombas et al. [12]	Spain	2020	Retrospective	661	16G	2 cores	No data
Hogan et al. [28]	United States	2020	Prospective	160	16G (54%) 18G (44%)	From 1 to 4 cores (Unknown)	No differences
Leclerc et al. [29]	Canada	2020	Retrospective	413	16G (98%) 18G (2%)	2 cores (86%) 1 core (14%)	No data
Sousanieh et al. [30]	United States	2020	Prospective	592	14G (57%) 16G (43%)	From 1 to 3 cores (2.2–2.3)	No data
Waldo et al. [31]	United States	2009	Prospective	162	14G	No data	No data
Pendon-Ruiz de Mier et al. [32]	Spain	2014	Prospective	241	16G	From 1 to 2 cores (unknown)	No differences
Li et al. [33]	China	2018	Retrospective	551	16G or 18G (unknown)	From 1 to 4 cores (unknown)	No differences
Lees et al. [34]	UK	2017	Retrospective	2563	16G	From 1 to 3 cores (unknown)	No data
Torres et al. [35]	Mexico	2011	Retrosepctive	623	16G	No data	No data
Current study	Spain	2021	Retrospective	221	16G	1 core (3.2%) 2 cores (28.1%) 3 cores (68.8%)	More complications in patients with one kidney core obtained.

## Supplementary Materials

The following supporting information can be downloaded at: https://www.mdpi.com/article/10.3390/jcm11051459/s1, Table S1: Multivariate logistic multinomial regression analysis for independent risk.
