# Supplementary material for: Safety of Obtaining an Extra Biobank Kidney Biopsy Core
Source: J Clin Med. 2022 Mar 7;11(5):1459. doi: 10.3390/jcm11051459 (PMC8911133; doi:10.3390/jcm11051459)
Supplement: Supplementary file 1 [file jcm-11-01459-s001.zip › jcm-1616893-supplementary.pdf]

Supplementary Table S1: Multivariate logistic multinomial regression analysis for independent risk factors of presenting complications after kidney biopsy.

Variable	OR	CI (95%)	Lateral significance (p)
PT (seconds)*	1.61	1.04-2.49	**0.035**
Renal cores (1 core vs 2/3 cores)	7.46	1.28-43.64	**0.026**
Age (years) *	1.01	0.98-1.03	0.67
Platelets (n) *	1	1-1	0.258
Weight (Kg) *	1.02	0.99-1.05	0.245
Hb pre (gr/dL)*	1.05	0.86-1.29	0.625
Dependent variable: Minor complications vs Absence of complications			
PT (seconds)*	1.06	0.58-1.95	0.857
Renal cores (1 core vs 2/3 cores)	5.93	0.35-99.69	0.217
Age (years) *	1.03	0.99-1.07	0.186
Platelets (n) *	1	1-1	0.257
Weight (Kg) *	1.01	0.96-1.06	0.785
Hb pre (gr/dL)*	1.44	0.95-2.18	0.082
Dependent variable: Major complications vs Absence of complications			

Dependent variable: Complications after kidney biopsy (minor complications *vs* major complications *vs* absence of complications).

PT: Prothrombin time, *Hb pre*: Haemoglobin level pre-KB, *CI:* Confidence Interval

* Quantitative variables included in the multivariate logistic binary regression model.
